# Silicon Nanowire Photocathodes for Photoelectrochemical Hydrogen Production

**DOI:** 10.3390/nano6080144

**Published:** 2016-08-05

**Authors:** Soundarrajan Chandrasekaran, Thomas Nann, Nicolas H. Voelcker

**Affiliations:** 1 Future Industries Institute, University of South Australia, South Australia 5095, Australia; soundarrajan.chandrasekaran@mymail.unisa.edu.au; 2 MacDiarmid Institute for Advanced Materials and Nanotechnology, Victoria University of Wellington, Wellington 6140, New Zealand; thomas.nann@vuw.ac.nz

**Keywords:** silicon nanowire, nanostructure, anti-reflective surface, bioinspired catalyst, hydrogen production

## Abstract

The performance of silicon for water oxidation and hydrogen production can be improved by exploiting the antireflective properties of nanostructured silicon substrates. In this work, silicon nanowires were fabricated by metal-assisted electroless etching of silicon. An enhanced photocurrent density of −17 mA/cm^2^ was observed for the silicon nanowires coated with an iron sulphur carbonyl catalyst when compared to bare silicon nanowires (−5 mA/cm^2^). A substantial amount of 315 µmol/h hydrogen gas was produced at low bias potentials for the silicon nanowires coated with an iron sulphur carbonyl catalyst.

## 1. Introduction

The depletion of fossil fuels and the effects of global warming and pollution caused by their combustion is a major challenge of modern societies. In recent times, hydrogen (H_2_) production and storage has been investigated as a potential renewable energy concept. By mimicking the process of natural photosynthesis by plants, an artificial photosynthesis device can be constructed. An artificial photosynthesis device consists of a photoanode and a photocathode, respectively, for the oxidation and reduction of water molecules to O_2_ and H_2_, respectively [1].

Suitable photoelectrodes for photoelectrochemical (PEC) water splitting are titanium dioxide (band gap 3.0 eV), cadmium selenides (band gap 1.7 eV), cadmium sulphides (band gap 2.4 eV) and silicon (band gap 1.1 eV) [2,3]. Silicon is considered to be optimal photoelectrode because of its low band gap, abundant availability in the earth’s crust and broad solar absorption spectrum leading to high solar energy conversion efficiency [4,5]. Nanostructured forms of silicon namely porous silicon (pSi) [6,7], pSi micro/nanoparticles [8,9,10] and silicon nanowires (SiNWs) [11,12,13] have demonstrated its potential in solar water splitting. The features demonstrating the potential of SiNWs includes the tunable band gap and antireflective surfaces [14,15,16,17,18,19,20,21,22]. A reproducible and well-regulated fabrication of SiNWs can be achieved through a metal-assisted electroless chemical etching (MACE) approach [23]. High surface area pSi can also be fabricated using MACE which has inward pores instead of vertically standing wires [24,25]. Well-ordered silicon pores/wires can be fabricated using a lithographical etching technique [26,27]. Both SiNWs and pSi have been widely used as photoelectrodes, in PEC water splitting. However, in order to achieve an improvement in the proton reduction using SiNWs/pSi, an electrocatalyst is required. 

Platinum (Pt) is an excellent electrocatalyst for proton reduction, but its scant availability in the earth’s crust and its high price limits widespread use [28]. A non-noble earth abundant electrocatalyst would be an alternative to overcoming this issue and help in the up-scaling of solar water splitting technologies [29,30].

For SiNWs coated with Pt nanoparticles (NPs), photocurrent densities of −20.7 mA/cm^2^ (*p-*type silicon) [31] and ~25 mA/cm^2^ (0 V vs. Pt counter electrode, *n*-type silicon) were achieved [12]. Similarly, *p-*type SiNWs coated with more abundant molybdenum sulphide (MoS_3_) catalyst showed a photocurrent density of −24.9 mA/cm^2^ [32]. In the case of *p-*type pSi fabricated using MACE coated with Pt NPs, a photocurrent density of ~−22.5 mA/cm^2^ was reached [25]. The conditions used to measure current densities for the above reports were 0 V vs. RHE and light intensity (100 mW/cm^2^) unless otherwise stated. More recently, we have fabricated a pSi photocathode using an electrochemical anodisation technique for solar hydrogen production [7] where in the presence of indium phosphide nanocrystals (InP NCs) and iron sulphur carbonyl (Fe_2_S_2_(CO)_6_) a photocurrent density of −1.2 mA/cm^2^ (light intensity 100 mW/cm^2^) at −500 mV bias potential was achieved [7].

In this work, we successfully demonstrated the fabrication of a SiNWs working electrode coated with Fe_2_S_2_(CO)_6_ catalyst. The fabricated working electrode was assembled into a three electrode electrochemical cell for photocurrent density measurements and the H_2_ produced in the headspace was analysed by gas chromatography (GC).

## 2. Results and Discussion

**Figure 1:**
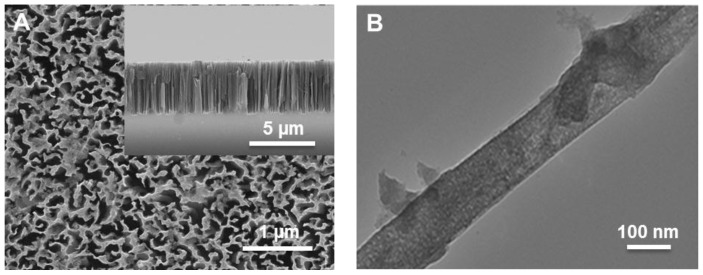
(**A**) Scanning electron microscopy (SEM) image of the fabricated silicon nanowires (SiNWs) for 10 min of etching time. The inset shows the cross-sectional SEM image; (**B**) transmission electron microscopy (TEM) image of an individual SiNW.

*p-*type planar silicon (resistivity 10–20 mΩ·cm) was etched by means of the MACE technique with 10 min of etching time. Figure 1A show a representative top-view scanning electron microscopy (SEM) image of etched SiNWs sample (10 min) and the inset show the corresponding cross-sectional view. The length of the SiNWs was measured to be approximately 4 µm. Figure 1B shows the transmission electron microscopy (TEM) image of an individual SiNW with a diameter of approximately 120 nm.

**Figure 2:**
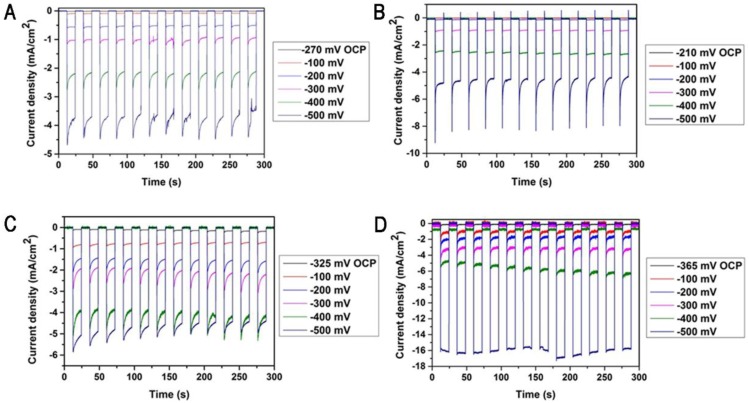
Current density measurements for the planar silicon (**A**), bare SiNWs (**B**), planar silicon coated with Fe_2_S_2_(CO)_6_ catalyst (**C**) and SiNWs coated with Fe_2_S_2_(CO)_6_ catalyst (**D**).

Figure 2A–D shows the current density measurements as a function of time at different bias potentials for planar silicon, SiNWs, planar silicon coated with Fe_2_S_2_(CO)_6_ catalyst and SiNWs coated with Fe_2_S_2_(CO)_6_ catalyst photocathodes, respectively. The current density measurements were acquired by ramping the bias potential between −100 to −500 mV for 5 min under AM 1.5 solar illumination (one sun) in 0.1 M H_2_SO_4_. The bare SiNWs photocathode gave a current density of −5 mA/cm^2^ which is 1.3-fold greater than the planar silicon (−4 mA/cm^2^) at a bias potential of −500 mV. An enhanced current density of −17 mA/cm^2^ was observed for SiNWs photocathode coated with Fe_2_S_2_(CO)_6_ catalyst which is 4.3-fold greater than the planar silicon photocathode coated with Fe_2_S_2_(CO)_6_ catalyst (~−5 mA/cm^2^) at a bias potential of −500 mV. We hypothesise that the high surface area of the nanostructured SiNWs improved the catalyst loading and reduced the reflection of the incoming light for improved solar energy conversion.

Therefore, attachment of a bioinspired Fe_2_S_2_(CO)_6_ catalyst on SiNWs increased the current density to 3.4 and 4.3-fold higher when compared to the bare SiNWs and planar silicon, respectively. These results, obtained with a synthetic [FeFe]-hydrogenase mimic, compare well with similar work using hydrogenase enzymes on semiconductor electrodes [24,33]. Very recently, a [FeFe]-hydrogenase enzyme was directly adsorbed onto high surface area pSi fabricated through a MACE technique [24]. A current density of 1 mA/cm^2^ at a bias potential of −500 mV vs. Ag/AgCl in phosphate buffer (light intensity of 10 mW/cm^2^) was achieved. This current density is several orders lower than the performance of SiNWs described in this work.

**Figure 3:**
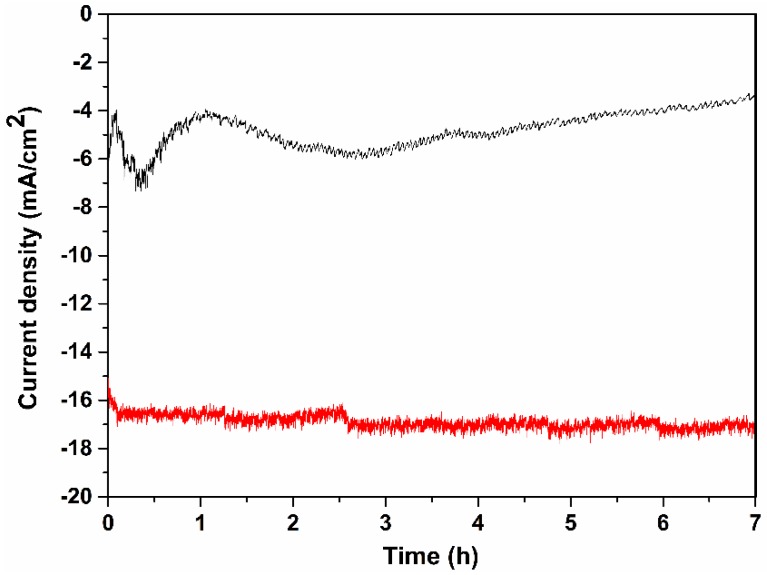
A long run measurement of planar silicon (black trace) and SiNW (red trace) coated with Fe_2_S_2_(CO)_6_ catalyst respectively in 0.1 M H_2_SO_4_ at a bias potential of −500 mV over 7 h (under illumination).

**Figure 4:**
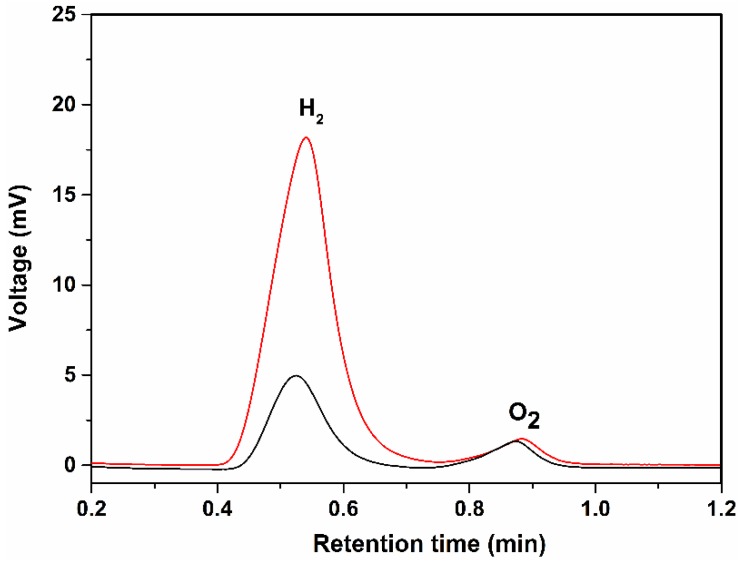
Gas chromatography (GC) analysis of the sample gas (500 µL) from the headspace of the photoelectrochemical (PEC) cell for planar silicon (black trace) and SiNW (red trace) coated with Fe_2_S_2_(CO)_6_ catalyst, respectively.

A long run measurement was performed to test the stability of the catalyst-loaded planar silicon and SiNWs photocathode for 7 h in 0.1 M H_2_SO_4_ at a bias potential of −500 mV (Figure 3). The planar silicon coated with catalyst (black trace) showed an unstable current density and then decreased with time presumably due to surface oxidation and catalyst being desorbed from the planar silicon surface. However, the current density of SiNWs coated with catalyst (red trace) was stable over 7 h. The sample gas from both the photocathodes were quantified by collecting 500 µL of the sample gas after 1 h from the headspace of the PEC setup and injected into the GC. The peak value at ~0.5 min from the GC (Figure 4) confirmed the presence of H_2_ gas in both the photocathodes [34]. The peak value at ~0.9 min from the GC (Figure 4) corresponds to the atmospheric O_2_. The amount of H_2_ gas was determined to be approx. 315 µmol/h for SiNWs coated with catalyst (red trace) which is 4-fold greater than the planar silicon coated with catalyst (~85.7 µmol/h, black trace).

## 3. Materials and Methods

### 3.1. Materials

SiNWs were fabricated from *p-*type silicon wafers (Czochralski, Silicon Quest Intl. Ltd., San Jose, CA, USA) with resistivity of 10–20 mΩ·cm, orientation (100). Hydrofluoric acid (HF) (48%) was purchased from Scharlau Chemie (Chem-Supply Pty. Ltd. Australian representation, South Australia, Australia). Silver nitrate (AgNO_3_) and hydrogen peroxide (H_2_O_2_) (30%) were purchased from Merck (Victoria, Australia).

### 3.2. SiNW Fabrication

A *p-*type silicon wafer with resistivity of 10–20 mΩ·cm was used as a starting wafer. The wafer was cleaned by ultrasonication in acetone, ethanol and deionised water for 5 min, respectively. The wafers were cut into 1 × 1 cm^2^ pieces and dipped in 1:1 HF and ethanol solution to remove the native oxide layers. The unpolished side of the wafers were masked using sticky tape to avoid etching of the surface. Firstly, the wafers were dipped in 4.8 M HF and 0.02 M AgNO_3_ solution for 30 s to deposit Ag on the polished side. The wafers were then immediately dipped in the etching solution of 4.8 M HF and 0.1 M H_2_O_2_ for 10 min. The etched wafers were then rinsed with de-ionised water and the sticky tape was removed from the unpolished side of the wafers. Finally, the etched wafers were dipped in concentrated nitric acid for 20 min to remove Ag coating, then washed with de-ionised water.

### 3.3. Electrode Fabrication

The 1 × 1 cm^2^ planar silicon and SiNW array pieces were dipped in 1:1 HF:ethanol solution for 2 min to remove the native oxide layers. It was then dried in a stream N_2_ gas and quickly transferred to an argon purged glove box. Then, five layers of Fe_2_S_2_(CO)_6_ catalyst in toluene were drop casted on the samples at room temperature. A back contact to the samples (unpolished surface) was formed using In-Ga eutectic applied via a cotton swab. A copper plate was used as an electrical contact. 

### 3.4. Surface Characterisation

SEM images were obtained on a FEI Quanta 450 environmental scanning electron microscope (Hillsboro, OR, USA). TEM images were obtained on a computer-controlled TEM JEM-2100F (Jeol Pty Ltd., Peabody, MA, USA), equipped with a field emission gun. SiNWs were scrapped from the etched wafer and were suspended in ethanol. The samples were dried on a 300 lines/mesh copper grid coated with a Formvar film (PST ProSciTech, Queensland, Australia). The instrument was operated at a 200 kV accelerating voltage and images were acquired with a Gatan Orius SC1000 CCD camera (Pleasanton, CA, USA) mounted at the bottom of the column.

### 3.5. Photocurrent and GC Measurements

We used an Abet solar simulator (air mass 1.5–1 sun) to irradiate the samples. The solar simulator was calibrated against a silicon solar cell (New-Spec). Electrochemical measurements were then performed using a PG 310 potentiostat from HEKA Electronics (Lambrecht/Pfalz, Germany). For electrolysis a sealed three-electrode Teflon PEC cell was used consisting of a Pt counter electrode with a frit, a Ag/AgCl 3 M KCl reference electrode and the working electrode. The working electrode was illuminated with a light intensity of 100 mW/cm^2^ with 12 s dark and 12 s light cycle to measure the photocurrents over a period of 5 min. The potential between the working and reference electrodes was ramped between 0 and −500 mV in 100 mV steps. Photocurrent runs over 7 h were performed under a light cycle at a bias voltage of −500 mV. Gas in the headspace (500 µL) above the electrolyte was sampled after 1 h of electrolysis and analysed using a SRI 310C series GC (Torrance, CA, USA) equipped with a thermal conductivity detector and a column held at 70 °C in N_2_ as the carrier gas.

## 4. Conclusions

SiNWs coated with catalyst showed a 3.4-fold increase in the current density when compared to bare SiNWs. We demonstrated a strategy of fabricating a robust SiNW array photocathode using a bio-inspired catalyst that was able to consistently produce H_2_ gas for up to 7 h. The attachment of hydrogenase synthetic mimics as catalysts onto high surface area SiNW photocathodes initiate an affordable solar energy conversion by replacing Pt-based catalysts.
